# Vitamin C as a treatment for organ failure in sepsis

**DOI:** 10.1186/s40001-023-01183-7

**Published:** 2023-07-05

**Authors:** Zitong Wang, Liang Liu, Lixia Liu

**Affiliations:** 1 Department of Critical Care Medicine, The Fourth Hospital of Hebei Medical University, Shijiazhuang, 050000 China; 2 Institute of Oncology, The Fourth Hospital of Hebei Medical University, Shijiazhuang, China

**Keywords:** Vitamin C, Sepsis, Organ failure, Pathogenesis, Pharmacological mechanism

## Abstract

Sepsis is defined as life-threatening organ dysfunction caused by a dysregulated host response to infection, with a high morbidity and mortality rate. Exogenous vitamin C supplementation is a potential therapeutic option for the treatment of multi-organ dysfunction in sepsis due to the significantly lower levels of vitamin C in the circulating blood of sepsis patients compared to healthy subjects and the importance of vitamin C in many of the physiological processes of sepsis. Vitamin C may influence the function of numerous organs and systems, including the heart, lungs, kidneys, brain, and immune defences, by reducing oxidative stress, inhibiting inflammatory factor surges, regulating the synthesis of various mediators and hormones, and enhancing immune cell function. With the development of multiple clinical randomized controlled trials, the outcomes of vitamin C treatment for critically ill patients have been discussed anew. This review's objectives are to provide an overview of how vitamin C affects various organ functions in sepsis and to illustrate how it affects each organ. Understanding the pharmacological mechanism of vitamin C and the organ damage caused by sepsis may help to clarify the conditions and clinical applications of vitamin C.

## Introduction

Sepsis is a serious and severe disease syndrome caused by an infectious injury. Globally, sepsis has a very high incidence, mortality, and disability rate. According to statistics, the annual incidence of sepsis can be as high as 60 million people, while the number of deaths exceeds 3 million. The causes of organ dysfunction in sepsis are complex, and different organs and systems can interact with one another to worsen the damage. For example, microcirculatory perfusion disorders can result in tissue hypoxia and the accumulation of toxic waste products from tissue metabolism, which in turn can cause endothelial dysfunction, increased vascular permeability and vascular leakage. Multiple organs throughout the body are affected, including increased pulmonary vascular permeability, decreased gas space in the alveoli, and a thickened barrier between the alveoli and blood vessels, all of which affect ventilation and diffusion function. To date, our treatment of organ damage in sepsis has been mostly supportive in nature, and we currently need to link the pathophysiological changes in each organ damage to the mechanisms of therapeutic agents to improve the understanding of this disease and its current treatment options.

Regarding the pathogenesis of sepsis, it is generally accepted that sepsis is an uncontrolled host response to pathogens that can manifest as multiple pathophysiological changes, such as imbalance of systemic inflammatory response and imbalance of immune regulation. Among these pathophysiological changes, imbalance of redox response plays a crucial role in the pathogenesis of sepsis. In recent years, there has been increasing experimental evidence of the important role of oxidative stress in the pathogenesis of sepsis. In an early investigation, Takeda et al. showed increased lipid peroxidation and decreased antioxidant levels in sepsis patients [1]. Quinlan et al. demonstrated that patients with septic ARDS have higher mortality and experience higher levels of oxidative stress and damage than patients with non-septic ARDS [2]. Sepsis causes a transition in redox homoeostasis to a pro-oxidant state, leading to an imbalance manifested by excessive production of reactive oxygen species (ROS), mitochondrial dysfunction, and collapse of the antioxidant system. ROS are generally oxidants generated by mitochondrial respiration and mitochondrial electron transfer. ROS induce post-translational changes of proteins, hence modifying their function and influencing cell signaling, gene expression, oxygen sensing, and other physiological functions. At low concentrations, ROS promote cell viability by enhancing antioxidant responses via activation of nuclear factor-erythroid 2-related factor 2. ROS are essential for bodily homeostasis and defense, but they can be harmful if they are produced in large amounts to overwhelm antioxidant defense system. ROS can trigger reversible or permanent damage to proteins, lipids, and nucleic acids in this setting, leading to endothelial dysfunction, cellular damage, and multi-organ dysfunction. In addition, ROS-induced damage to the glycocalyx and cell–cell junctions results in increased permeability, adhesion of leukocytes and platelets, and local activation of inflammation and coagulation, which ultimately results in impaired vascular reactivity and reduced vascular response to vasoconstrictors. Persistent systemic hypotension, increased vascular leakage and microcirculatory stasis increase oxygen diffusion distances, thereby exacerbating tissue cell hypoxia and possibly exacerbating cellular damage and organ failure.

Mitochondria are the primary source of explosive ROS production, and the massive accumulation of ROS and collapse of the antioxidant system causes further damage to mitochondria's respiratory and electron transport chains. The damaged mitochondria have trouble completely utilizing oxygen, resulting in insufficient energy supply and accumulation of metabolites, which exacerbate cell and tissue damage once more. In this process, mitochondria are both the initiators of oxidative stress and the targets of oxidative damage. Consequently, protection against mitochondrial function is crucial, and it may be an essential method for preventing the progression of oxidative stress and organ damage in sepsis.

Targeting medicines associated to redox abnormalities, such as antioxidants and ROS scavengers, is anticipated to improve the management of septic patients in the treatment of sepsis. Vitamin C (ascorbic acid) is the antioxidant most investigated in sepsis. Numerous organs and systems can benefit from the antioxidant and anti-inflammatory properties of vitamin C. In addition, Vitamin C participates in numerous physiological and pathological processes, particularly the incidence and progression of sepsis. Based on the fundamental mechanism of various organ injuries produced by sepsis, this review discusses the likely mechanism of vitamin C's effect on various organ functions in sepsis, as well as the present research status of vitamin C application in various organ injuries.

### Role of vitamin C: in sepsis-induced cardiomyopathy

Sepsis cardiomyopathy is a consequence of sepsis-related cardiovascular failure, and its severity is correlated with morbidity and death, making it a major cause of organ dysfunction in sepsis. Reduced left ventricular (LV) contractility and eventual LV dilatation, with or without right ventricular failure, characterize sepsis-induced cardiomyopathy.

#### Mechanisms

Current main studies indicate that septic cardiomyopathy is related with infection-induced inflammatory responses, oxidative stress, mitochondrial dysfunction, and calcium homeostasis imbalance. During the progression of sepsis, pathogen-associated molecular patterns (PAMPs) and damage-associated molecular patterns (DAMPs) bind to pattern recognition receptors (PRRs) and activate the corresponding signaling pathways, resulting in the production of a significant number of cytokines. Toll-like receptors (TLRs) are classical pattern recognition receptors that are widely distributed in vivo and present on cardiomyocytes. When Toll-like receptors are activated, cytokine production such as TNF-, IL-1, and iNOS (inducible nitric oxide synthase) increases, and activation of caspases increases, which all directly affect cardiomyocyte contractility and impair left ventricular ejection [3]. In addition, the binding of IL-1, TNF-, bacteria, viruses, or fungi to their respective receptors can activate NF-κB pathways. NF-κB activates the corresponding target genes, which leads to the creation of significant quantities of cytokines such as TNF-, IL-1, and IL-6. Large levels of cytokine-activated iNOS in addition to iNOS produced by cardiomyocytes result in considerable changes in circulating nitric oxide (NO). Low levels of NO modulate vascular tone via the cGMP pathway, limit oxidative damage, inhibit leukocyte and platelet adherence to the endothelium, and enhance cardiac contractility under normal conditions. In sepsis, however, nitric oxide metabolism is dysregulated, resulting in altered cardiac pre and afterload and decreased myocardial contractility [4, 5]. Additionally, NO enhances mitochondrial permeability.

The dysfunction of mitochondria is one of the major causes of septic cardiomyopathy. During sepsis, mitochondria endured various physical and chemical damages, such as hypoxia, hyperglycemia, reperfusion, etc., which led to the transformation of the body’s energy metabolism pathway to aerobic glycolysis and reduced the activity of mitochondrial respiratory chain complexes I, III, and IV. The above disordered mitochondrial function and produced a large number of ROS and reactive nitrogen species (RNS).

With the opening of calcium channels in the cardiomyocyte membrane, which allows a large amount of calcium to flow inward to the cytoplasm, mitochondrial permeability transition pore (mPTP) opens due to calcium overload, which allows mitochondrial genome (mtDNA) and cytochrome C to enter the cytoplasm and accelerates apoptosis in cardiomyocytes.

In addition, complement and DAMP can induce electrophysiological alterations in calcium-overloaded myocardium via endoplasmic reticulum stress, resulting in mitochondrial dysfunction [4].

#### Vitamin C therapy for sepsis-induced cardiomyopathy

Vitamin C possesses powerful antioxidant and anti-inflammatory properties. Vitamin C inhibits nuclear factor B activation, which in turn inhibits myocardial inflammatory cytokine signaling, thereby reducing the series of responses of NF-κB activation of the corresponding target genes, decreasing caspase activity, reducing cytokine and ROS production from upstream, and alleviating sepsis-related contractile dysfunction [6]. Vitamin C also inhibits ROS formation by inhibiting nicotinamide adenine dinucleotide oxidase, a crucial enzyme involved in ROS activation and synthesis [8]. In addition, through a series of electron-donating actions, vitamin C can exert potent antioxidant and mitochondrial-protective effects. First, vitamin C can directly provide electrons to peroxides, peroxynitrite (ONOO^−^), super negative oxygen ions, and hydroxyl radicals in order to directly scavenge ROS in vivo, mitigate the effects of large amounts of reactive oxygen species on the mitochondrial respiratory chain, and reduce energy supply and utilization barriers [7]. Second, vitamin C supplies electrons to other ROS scavengers, such as glutathione and vitamin E (α-tocopherol), and stimulates their activation, so increasing other antioxidant pathways that reduce organelle and cardiomyocyte oxidative damage. In addition, vitamin C inhibits the activation of nuclear factor B and decreases the release of mitochondrial cytochrome c, hence reducing cardiomyocyte apoptosis.

Existing research has shown that supplementing isolated cardiomyocytes with ascorbic acid during hypoxia/reoxygenation increases their resistance to cell death [9]. The treatment of singlet oxygen-exposed cardiomyocytes with vitamin C or vitamin E decreased the number of hypercontracted cardiomyocytes, and ascorbic acid provided considerable antioxidant protection against isolated cardiomyocytes [10]. This indicates that antioxidant vitamin therapy may be a viable treatment option for septic cardiomyopathy.

### Role of vitamin C: in circulatory dysfunction

Acute circulatory failure caused by septic shock or sepsis is accompanied by pathophysiological changes in the heart, blood arteries, and a range of tissues and cells. Vascular injury is mostly indicated by vasomotor dysfunction, endothelial dysfunction, and increased endothelial permeability, whereas cardiac injury is primarily manifested by lower myocardial contractility and depressed cardiac function. Previously, the loss in cardiac function owing to septic cardiomyopathy was described; this part will provide an overview of vascular injury.

#### Mechanisms

Vascular tension is altered in sepsis, and it is believed that increased generation of nitric oxide, activation of potassium channels, and downregulation of adrenergic receptors are responsible for vasodilatory dysfunction. Septic damage is characterized by alterations in vasodilatory tone, decreased vasopressor response, and impaired myocardial function, which are caused by elevated NO levels.

In sepsis, endothelial dysfunction is one of the signs of vascular damage. On the endothelium surface of blood arteries are endothelial cells composed of vascular endothelial cells, intercellular junctions, and glycocalyx groups. Sepsis affects nearly all aspects of endothelial cell (EC) function, which is a major component in the progression of sepsis to organ failure [12]. Endothelial cells dynamically modulate vascular tone, vascular barrier, coagulation pathways, and leukocyte adhesion under steady-state settings. Endothelial cells undergo various phenotypic and functional changes during sepsis and acute inflammation, beginning with an adaptive response endothelial shift toward promoting a pro-inflammatory, pro-coagulant, and pro-adhesive phenotype. As inflammatory variables grow and redox equilibrium becomes unbalanced, excessive levels of reactive oxygen and reactive nitrogen species cause breakdown of the polysaccharide envelope, cell death, increased permeability, and reduced vascular responsiveness, resulting in an organism-harming response. Thus, in sepsis, endothelial cells are a primary source of oxidative stress creation and inflammation promotion, as well as a key target of oxidative stress and inflammatory damage [11]. Multiple clinical studies have demonstrated a link between oxidative stress, mitochondrial dysfunction, and the severity of septic shock.

#### Vitamin C therapy for circulatory dysfunction

Vitamin C can reduce vascular damage in sepsis by enhancing vascular responsiveness, preserving the endothelium barrier, acting as an antioxidant, and decreasing inflammation.

Ascorbic acid protects arterial reactivity through the inhibition of endothelial cell-induced nitric oxide synthase expression and increased NO bioavailability, thereby reducing nitric oxide expression in endothelial cells and inhibiting intractable vasodilation in small arteries [13]; on the other hand, vitamin C is an essential cofactor for the synthesis of endogenous catecholamines such as norepinephrine, dop Vitamin C improves poor vascular reactivity and hemodynamic state while increasing adrenergic transmission. In rats and mice CLP (cecum ligation and puncture) models, ascorbic acid infusion inhibits arterial and blood pressure responses to vasoconstrictors, norepinephrine, and angiotensin II [13, 14]. Lavillegrand et al. found that intravenous vitamin C treatment (40 mg/kg) reduced capillary refill times in fingertips and knees, and the central to peripheral temperature gradients, and dramatically enhanced microvascular reactivity [16].

Ascorbic acid is capable of tightening the endothelium barrier via several mechanisms. [15] Constitutive endothelial nitric oxide (eNO) controls endothelial permeability and avoids the loss of tight intercellular connections. Ascorbic acid treatment of endothelial cells preserves eNO production via eNOS (endothelial nitric oxide synthase) and decreases endothelial permeability. Ascorbic acid inhibits the activity of protein phosphatase 2A (PP2A), which dephosphorylates closure proteins, to limit vascular leakage. The phosphorylation of closure proteins is necessary for maintaining tight junctions [17]. Zhou et al. administered ascorbic acid (200 mg/kg i.v.) to CLP mice and demonstrated that vitamin C inhibits vasodilation by inhibiting iNOS and nitric oxide synthase to reduce NO synthesis; and that vitamin C maintains closure protein phosphorylation by inhibiting activation of protein phosphatase 2A, thereby tightening the vascular endothelium, which reduces vascular leakage [21]. In addition, ascorbic acid reduces microcirculatory blood flow damage by inhibiting the production of intracellular adhesion molecule (ICAM) generated by tumor necrosis factor [19].

Ascorbic acid protects endothelial cells from oxidative injury. Nitric oxide metabolite (NO_X_) is the primary generator of ROS in endothelial cells and cardiomyocytes. Wilson et al. showed that the addition of ascorbic acid to endothelial cells inhibited p47phox subunit expression, hence preventing NO_X_ activation [18]. Ascorbic acid decreases oxidative stress in endothelial cells by lowering intracellular O_2_ generation, which is caused by NO_X_ activation. Inhibiting the Jak2/Stat1/IRF signaling pathway, ascorbic acid lowers the generation of superoxide (O_2_^−^), hydrogen peroxide, and peroxynitrite. Ascorbic acid protects oxidative stress-induced pathological vasoconstriction and loss of the endothelium barrier by preventing the oxidation of tetrahydrobiopterin (BH4), a cofactor of eNOS. eNO has a vital function in endothelium-dependent vasodilation. eNO avoids the depletion of eNO and dissociation of eNOS by promoting solubility in smooth muscle cells. vasodilation is initiated by guanyl cyclase and raising cyclic guanosine monophosphate. These various activities explain vitamin C’s pressor impact in a vasodilatory shock model [20].

### Role of vitamin C: in sepsis-induced acute lung injury

Acute respiratory distress syndrome (ARDS) is a fatal complication of severe sepsis. Patients with sepsis can progress rapidly to ARDS and is associated with inpatient mortality.

#### Mechanisms

Severe diffuse alveolar destruction is the major histological feature of ARDS, which is typically driven by localized inflammation and endothelial dysfunction. According to recent research, vascular endothelial (VE) cell injury, alveolar epithelial cell death, inflammatory response, oxidative stress, and compromised epigenetics are the main causes of septic ARDS. Various injury factors (e.g., bacterial and viral attack) can directly or indirectly cause various responses in the body, manifested by activation of alveolar macrophages, accumulation of neutrophils and monocytes, release of large amounts of pro-inflammatory mediators and chemokines (e.g., TNF, IL-6, and IL-8), platelet activation, and microthrombosis, which can eventually lead to damage to distal alveolar structures and associated microvascular areas.

#### Vitamin C therapy for sepsis-induced acute lung injury

In sepsis-induced ARDS, vitamin C improves pulmonary epithelial barrier function. To regulate alveolar fluid clearance, vitamin C increases the transcriptional expression of water channel protein 5, epithelial sodium channel (ENaC), and Na^+^/K^+^ ATPase. The intravenous administration of 200 mg/kg vitamin C to animals with endotoxin-lipopolysaccharide-induced sepsis decreased protein concentration in bronchoalveolar lavage fluid and diminished pulmonary edema [22]. Vitamin C enhances the endothelial glycocalyx barrier in order to protect the endothelium barrier. Serum multiligand proteoglycan syndecan-1, a biomarker of glycocalyx integrity, is an essential structural component of the glycocalyx that lines the endothelial surface of vascular lumens including alveolar capillaries. In sepsis, several damage causes lead to breakdown of the glycocalyx barrier, and plasma syndecan-1 is greatly raised, which is one of the earliest and most critical signs of ARDS. Qiao et al. discovered in critically sick patients with septic ARDS that high-dose vitamin C (50 mg/kg administered every 6 h for 96 h) lowered plasma syndecan-1 levels in ARDS patients for 48 h, which was strongly correlated with changes in oxygen saturation and all-cause mortality at 28 days [23]. Vitamin C also maintains the integrity of the intercellular skeletal structure of alveolar epithelial cells, lowers the production of pro-inflammatory mediators and neutrophil capture in the lung, and enhances pulmonary organ function. In a study by Fisher et al., intraperitoneal injection of 200 mg/kg vitamin C into mice with acute lung injury induced by intraperitoneal injection of mouse fecal fluid restored Na–K-ATPase activity and induced tight junction protein claudin-18 (CLDN18.2), the closure protein occluding, and the tight junction protein zona occludens-1 (ZO-1) expression, thereby restoring the integrity of the. Vitamin C decreased IL-8 and MCP-1 mRNA expression, blocked monocyte adherence in endothelial cells, reduced the release of HMGB-1 from macrophages, and attenuated the pro-inflammatory response, according to Kang et al. HMGB-1 initiates a fatal inflammatory response by augmenting the release of pro-inflammatory molecules TNF, IL-1, IL-6, IL-8, and MIP-1β [25].

### Role of vitamin C: in sepsis-induced acute kidney injury

The kidney is one of the most susceptible organs affected by sepsis, resulting in sepsis-associated acute kidney injury (SA-AKI), a high incidence rate, and death.

#### Mechanisms

In sepsis, the pathophysiological processes of AKI are incompletely known. Due to the fact that renal hypoperfusion can result in renal hypoxia, it has been regarded as one of the leading causes of AKI in sepsis. In addition, microcirculatory dysfunction, the toxic effects of inflammatory mediators (cytokines, chemokines, and complement) on renal tubules, and metabolic reprogramming play crucial roles in the development of S-AKI. Furthermore, there is substantial evidence that the development of septic AKI is directly related with oxidative damage to renal tubular cells and renal tissue. Renal hypoxia and ischemia enhance the generation of ROS, which damage biomolecules and membranes, impair organelle function, and contribute to renal tubular cell injury, inflammation, and vascular dysfunction. Leukocyte recruitment, endothelial cell stimulation, nitric oxide production, and mitochondrial dysfunction all contribute to elevated ROS generation and oxidative damage throughout the progression of AKI.

#### Vitamin C therapy for sepsis-induced acute kidney injury

The water-soluble, low-molecular-weight, non-protein antioxidant ascorbic acid is a promising drug for the treatment of renal injury in critically ill patients. Vitamin C in the blood can prevent oxidative kidney damage by interacting with free radicals, decreasing lipid peroxidation damage, and protecting vitamin E, so playing an important protective role.

Vitamin C inhibits microvascular dysfunction and inflammation in septic rats by blocking the activation of necrosis factor B by tumor necrosis factor. In addition, ascorbic acid is absorbed by endothelial cells and boosts endothelial nitric oxide synthase, improves nitric oxide bioavailability, and enhances kidney perfusion and oxygenation. Moreover, sodium ascorbate promotes a decrease in tubular sodium reabsorption to decrease oxygen consumption and adds to the enhancement of renal PO_2_. In a sheep septic AKI model, Fenhammar et al. discovered that administration of mega-doses of sodium ascorbate reversed renal medullary tissue hypoperfusion and tissue hypoxia and significantly improved microcirculatory impairment, which correlated with complete reversal of AKI, as indicated by urinary flow rate and significant improvement in creatinine clearance and effective improvement of plasma creatinine levels back to normal [26]. Marik et al. found, in a retrospective controlled study of 47 patients with severe sepsis or infectious shock on vitamin C, vitamin B1 and hydrocortisone, that only 10% of patients with acute kidney injury in sepsis on vitamin C, vitamin B1 and hydrocortisone required renal replacement therapy, which was significantly lower than in sepsis patients who did not apply vitamin C or vitamin B1 [27].

### Role of vitamin C: in sepsis-associated encephalopathy and cognitive impairments

When sepsis produces acute neurological impairment, it is called sepsis-associated encephalopathy (SAE). Studies have indicated that 8% to 70% of sepsis patients experience SAE. SAE is a broad brain damage caused by an internal illness in the absence of a detectable CNS (central nervous system) infection. In sepsis and infectious shock, the CNS becomes vulnerable due to the collapse of the blood–brain barrier and the ensuing inflammatory and neurotoxic processes; thus, patients may present with varied degrees of brain dysfunction, ranging from confusion and delirium to coma.

#### Mechanisms

Regarding the pathogenesis of sepsis-induced brain dysfunction, the widely accepted theory is that sepsis can damage the brain through a variety of processes, including impaired cerebral microcirculation, inflammatory response, imbalance related to neurotransmitters, impaired blood–brain barrier, oxidative stress, apoptosis, microglia activation and disturbed metabolic levels, which damage neurons disrupting normal brain function leading to cognitive impairment.

Compared to other organs, the brain is regularly exposed to higher quantities of free radicals and ROS due to its high metabolic rate. The brain may be one of the initial organs damaged by sepsis. ROS are created during the inflammatory response and promote lipid peroxidation in the brain. Lipid peroxidation leads to mitochondrial malfunction and cytochrome c release, which promotes neuronal death [28, 29] due to dysregulation of the redox system. Moreover, brain imaging studies indicate that regions of the brain exhibit symptoms of cerebral ischemia during sepsis. The existence of neuronal mitochondrial degeneration and neuronal apoptosis in multiple brain areas of sepsis humans and animal models [30, 31] has been reported. Sharshar et al. discovered that neuronal apoptosis in septic rats and patients may be related to increased production of iNOS and raised levels of NO and ONOO^−^, which disrupt the mitochondrial electron transport chain [32].

#### Vitamin C therapy for sepsis-associated encephalopathy and cognitive impairments

Ascorbic acid and a-tocopherol are the non-enzymatic antioxidants that have been investigated the most in septic brain damage. Vitamin C primarily scavenges and prevents the production of ROS. In addition, ASC may minimize damage to the blood–brain barrier by defending the endothelial barrier and preserving capillaries. The sodium-dependent vitamin C transporter protein 2 (SVCT2) transports vitamin C from the blood to the choroid plexus cerebrospinal fluid in the brain, where it is ultimately absorbed by neurons. ASC serves as both a neuroprotector and neuromodulator in the brain [33, 34].

In a mouse model of cecum sludge (CS) sepsis, Consoli et al. reported that the initial onset of sepsis was accompanied by a large fall in brain vitamin C levels and a significant rise in hepatic vitamin C production [35]. In a rat model of sepsis, it was discovered that vitamin C improved cognitive function and survival by reducing oxidative stress in brain tissue and inflammatory factor levels [36]. Nevertheless, Park et al. showed in a retrospective observational analysis that the combination of vitamin C and thiamine did not diminish the number of days of ICU delirium [37]. In this study, vitamin therapy was not randomized, and starting and discontinuing vitamin therapy was left to the discretion of the emergency room and intensive care unit physicians. Therefore, additional prospective trials are required to clarify the role of vitamin C in the therapy of sepsis-associated encephalopathy.

### Role of vitamin C: patients with sepsis

In recent years, vitamin C treatment has been the subject of clinical randomized controlled trials designed to improve multi-organ dysfunction and survival in critically ill patients. Fowler et al. began investigating the safety and dosing of high-dose IV vitamin C in a clinical setting with monotherapy and discovered a significantly faster decline in sequential organ failure assessment (SOFA) scores in the high-dose vitamin C group, decreased inflammatory markers in the vitamin C group and infection marker levels, and maintained endothelial injury marker levels [38]. In a retrospective analysis, Marik et al. discovered that intravenous hydrocortisone, vitamin C, and thiamine reduced in-hospital mortality and improved 72 h SOFA scores in patients with severe sepsis or viral shock [7]. The CITRIS- ALI study, published in JAMA in 2019, evaluated the importance of vitamin C therapy in patients with sepsis and ARDS. The treatment group received 50 mg/kg of vitamin C intravenously every 6 h for 96 h, showing a substantial reduction in 28 day mortality [39].

However, the results of a new phase 3, multicenter, randomized, controlled study of vitamin C to attenuate organ dysfunction (LOVIT) raise questions about the large-scale clinical use of vitamin C. The incidence of death or permanent organ failure at day 28 was higher in the vitamin C-treated group than in the control group [40]. The authors of the LOVIT trial were unable to provide an explanation for the unexpected outcomes. Potential explanations could be attributed to baseline imbalances in patient characteristics. In the vitamin C group, patients had higher lactate levels and more patients showed signs of shock, indicating that the septic patients in the vitamin C group appeared to have a more severe condition. Furthermore, it is imperative to conduct further evaluation of the therapeutic dosage of vitamin C, the duration of administration, and the potential coapplication of other antioxidants in clinical patients. These evaluations serve as essential prerequisites for enhancing the precision and efficacy of vitamin C utilization.

It is reassuring to note that several meta-analyses have provided reliable evidence for the beneficial effects of vitamin C in improving organ dysfunction. In a systematic review and meta-analysis of randomized clinical trials conducted by Martimbianco et al. [42] and published in Journal of Crit Care, it was found that vitamin C, compared to placebo, significantly reduced the all-cause mortality within 28 days (relative risk [RR] 0.60, 95% confidence interval [CI] 0.45–0.80, 4 RCTs, 335 participants) [41]. Additionally, a recent meta-analysis of a randomized controlled trial on the efficacy of intravenous vitamin C therapy in sepsis or septic shock patients, published in Critical Care in 2023, revealed that intravenous administration of vitamin C significantly improved the delta SOFA scores from baseline to 72–96 h and significantly reduced the duration of vasopressor use. Liang et al. also conducted a subgroup analysis of the dose and duration of vitamin C administration and found that doses between 25 and 100 mg/kg/d were associated with a significant reduction in mortality at 28 or 30 days. Furthermore, the continuous administration of vitamin C for 96 h and its combination with hydrocortisone and thiamine significantly improved the delta SOFA scores, while there was no statistically significant difference in reducing overall mortality [42].

Over the years, the focus of outcome indicators for vitamin C treatment of critically ill patients has been on improving all-cause mortality, yet mortality represents the ultimate clinical outcome measure. The current studies on mortality improvement remain controversial, but reliable findings indicate that intravenous vitamin C (IVVC) significantly expedites the decline in SOFA scores, as confirmed by trial sequential analysis (TSA). Further trials are unlikely to alter these conclusions and are therefore unnecessary [42]. Consequently, vitamin C treatment might be targeted to improve one or a few organs, and it is crucial to establish detailed organ-specific functional indicators and systemic markers for the more precise clinical application of vitamin C.

The effect of vitamin C on the organs is dependent upon the distribution and transport of vitamin C within the body, in addition to the unique tissue characteristics of each organ. Physiologically, vitamin C exhibits its highest concentration in the brain and adrenal glands, surpassing plasma levels. The high organ and intracellular ascorbic acid concentrations are mainly due to ascorbic acid uptake by SVCT1 (SLC23A1) and SVCT2 (SLC23A2). Vitamin C enters the adrenal medullary chromophobe cells through SVCT2 and acts as a cofactor for β-hydroxylase, facilitating the conversion of dopamine to norepinephrine, which is the underlying reason why the application of vitamin C in severe sepsis can reduce the use of vasopressor drugs. Consoli et al. demonstrated in a mouse model of sepsis that after 4 h of cecal slurry (CS) treatment, cerebral vitamin C levels decreased by approximately 10%. [35]. Therefore, intravenous supplementation of vitamin C plays a crucial role in preventing the acute decline of vitamin C levels in the brain caused by sepsis. Ascorbic acid has been shown to act as a spare/recycling α-tocopherol in the lipid bilayer, particularly in the lipid-rich environment of the brain, effectively preventing sepsis-induced lipid peroxidation [43]. Consequently, vitamin C may serve as a vital therapeutic approach for sepsis-associated encephalopathy, with its potential to improve sepsis-related brain complications surpassing its impact on the mortality rate.

### Outlook

Taken together, recent research suggests that vitamin C has potential in improving multi-organ dysfunction in sepsis. However, more clarity is needed regarding the specific clinical characteristics of patients who would benefit from vitamin C therapy, as the effectiveness may vary depending on the organs involved in sepsis. Furthermore, the appropriate dose of vitamin C and the duration of continuous administration need to be further explored and mapped out. The dose and periodicity of treatment may be important prerequisites for treatment outcome; if the dose is too high, vitamin C may exert pro-oxidative effects and exacerbate the risk of hypoglycemia; if the periodicity of treatment is insufficient, it may not be sufficient to translate into clinically meaningful differences.

## Conclusions

Given the antioxidant activity of vitamin C and its involvement in several biochemical and biological processes, as well as the significantly lower levels of vitamin C in critically ill patients, this provides justification for vitamin C supplementation. Animal studies have demonstrated that vitamin C administration protects against organ damage caused by sepsis, including damage to the heart, lung, kidney, and brain. Regarding the organ-protective effects of high-dose vitamin C in critically ill patients, reliable results suggest that IVVC significantly accelerates the decline in SOFA scores and shortens the duration of vasopressor use, but there is still controversy regarding the improvement in morbidity and mortality, and further clarification is needed on the clinical characteristics of septic patients benefiting from vitamin C treatment and more refined indicators of individual organ involvement. Large-scale retrospective studies and randomized controlled trials with strict criteria are warranted. There is also a need to determine the optimal dose and duration of vitamin C use in septic patients and the relationship between vitamin C blood levels and organ protection in vivo, so that critically ill patients can derive the maximum benefit from vitamin C administration.

## Data Availability

Not applicable.

## Abbreviations

ARDS: Acute respiratory distress syndrome
ROS: Reactive oxygen species
Nrf2: Nuclear factor-erythroid 2-related factor 2
LV: Left ventricular
PAMPs: Pathogen-associated molecular patterns
DAMPs: Damage-associated molecular patterns
PRRs: Pattern recognition receptors
TLRs: Toll-like receptors
iNOS: Inducible nitric oxide synthase
NF-κB: Nuclear factor-kappaB
NO: Nitric oxide
cGMP: 3',5'-Cyclic guanosine monophosphate
RNS: Reactive nitrogen species
mPTP: Mitochondrial permeability transition pore
mtDNA: Mitochondrial genome
ONOO^−^: Peroxynitrite
EC: Endothelial cell
CLP: Cecum ligation and puncture
eNO: Endothelial nitric oxide
eNOS: Endothelial nitric oxide synthase
PP2A: Protein phosphatase 2A
ICAM: Intracellular adhesion molecule
NO_X_: Nitric oxide metabolite
p47phox: 47-KDa neutrophil oxidase factor
Jak2/Stat1/IRF: Janus kinase2*/*signal transducer and activator of transcription 1/Interferon regulatory factor
O_2_^−^: Superoxide
BH4: Tetrahydrobiopterin
AQP5: Water channel protein 5
ENaC: Epithelial sodium channel
CLDN18.2: Claudin18.2
ZO-1: Zona occludens 1
MCP-1: Monocyte chemoattractant protein-1
HMGB-1: High mobility group box chromosomal protein-1
MIP-1β: Macrophage inflammatory protein-1alpha
SA-AKI: Sepsis-associated acute kidney injury
SAE: Sepsis-associated encephalopathy
SVCT2: Sodium-dependent vitamin C transporter protein 2
NADPH: Nicotinamide adenine dinucleotide phosphate
IVVC: Intravenous vitamin C
TSA: Trial sequential analysis
